# Is ISO20121 Certification a Detour or Gamechanger for Eco-Striving Sport Events? A Conceptual Typology

**DOI:** 10.3389/fspor.2021.659240

**Published:** 2021-05-13

**Authors:** Hans Erik Næss

**Affiliations:** Kristiania University College, Oslo, Norway

**Keywords:** environmental sustainability, formula E, organizational hypocrisy, bazaar economy, green clubs

## Abstract

An increasing number of sport organizations express interest in international accreditation standards as a way of tackling environmental sustainability (ES) challenges. This article, however, argues that the commercial context of these standards and “the bazaar economy” in which its suppliers operate may hamper the diverse solutions needed to reduce the ecological footprint of sport. At the same time, it acknowledges that standards are useful to certain sporting organizations. To sort out the pros and cons of the relation between such standards and ES work in sport organizations, the Formula E (for Electric) championship is used as case example. The championship became the first category in motorsport to receive ISO certification in 2018, and 2 years later, it achieved net zero carbon footprint from inception. On that basis, the article provides a typology that provides coordinates for empirical research on how sport organizations may avoid “organizational hypocrisy” in their ES work by viewing the pros and cons of ISO certification. While the practical implication is insight into what it takes for a sport organization to achieve a specific certification, the academic implication is conceptual coordinates for empirical and comparative research of ES initiatives and certifications.

## Introduction

Environmental sustainability (ES) and sport events have been coupled since the early 1990s. Partly due to different interpretations of ES (Chernuschenko, 1996, Paquette et al., 2011), as well as inconsistent applications of it by sport governing bodies (Geeraert and Gauthier, 2018), this has however led to a variety of initiatives but little homogenization of ES practice and assessment. For that reason, since the early 2000s, there has been a growing emphasis on developing international and generic *standards* for environmental performance at sport events (Mallen et al., 2010). In a broad sense, standards define normative rules on behavior that are voluntarily adhered to Timmermans and Epstein (2010) and Brunsson et al. (2012). However, given the problem of agreeing upon common measures in sport, authors warned that it could lead to reduction of credibility of environmental performance reports (Mallen et al., 2010; Vanwynsberghe, 2015; Collins and Cooper, 2017) or, in a more general sense, “organizational hypocrisy.” This term refers to organizations' inconsistency between talk, decisions, and action (Brunsson, 1989, 2006). Whereas, this inconsistency can be upheld for strategic reasons, i.e., to preserve legitimacy in the face of conflicting stakeholder demands (Cho et al., 2015), it can also become a delegitimizing process as failing to “walk the talk” may overshadow the engagement for ES issues in the first place.

This topic is central to studies that question the ES efforts from sport organizations (Petersson and Vamling, 2016; Johnson and Ehsan Ali, 2017; Malhado and Limdemberg, 2017; Miller, 2017; Geeraert and Gauthier, 2018; Popa, 2019; Dendura, 2020). Among the most vocal critics, we find Boykoff and Mascarenhas (2016) who argue that the IOC and its hosts have

capitalized on the sustainability zeitgeist without actually embracing significant environmental principles. This amounts to buying the eco-label through aggressive public-relations campaigns rather than forging meaningful environmental policies with positive material ramifications. In short, symbolism swallows substance (Boykoff and Mascarenhas, 2016, p. 2).

A case for sport organizations to address is therefore how organizational hypocrisy, related to ES work, may be reduced. Despite the focus on “certifications,” that is, the verification tasks performed by an independent auditing body to ensure that organizations adhere to sustainability standards, criticisms are plenty. It is not given that striving for standards represent the best eco-improving options for all forthcoming events or organizations. Some reasons are, on the one hand, cost, creativity, and credibility issues and, on the other, the certification system's reminiscence of “the bazaar economy” (Geertz, 1978). However, whereas Geertz portrays the bazaar economy as a contrast to a modern capitalist market, mostly because of money's role in the latter, this article follows Fanselow's (1990) argument that these arenas for selling and purchasing products and services are quite similar. Akin to how “market, brand names and trademarks act as classificatory devices by which the provenance of goods in capitalist markets becomes identifiable” (Fanselow, 1990, p. 253), there is a comprehensive folklore in bazaars about preferences and information exchange opportunities.

Most importantly, information in bazaars is “poor, scarce, maldistributed, inefficiently communicated, and intensely valued” (Geertz, 1978, p. 29). Similar to sporting organizations on the look for ISO costs or what it would take for a football club to become certified by one standard or another, the “search for information one lacks and the protection of information one has is the name of the game” (Geertz, 1978, p. 29). For example, when the Health, Safety and Environmental Manager of Manchester United FC, an ISO certified club, was asked if he had any advice to prospective ISO certificants, this is what he said: “make sure you get reputable and credible advice, guidance and support. Be very careful whom you approach^1^” As a consequence, the cases discussed in this article indicate that two procedures become central to customers in sport. The first is clientelization, which is the tendency for repetitive purchasers to establish continuing relationships with particular purveyors of them, rather than search widely through the market at each occasion of need' (Geertz, 1978, p. 30). Because trading “involves an ongoing search for specific partners, not mere offers of goods to the public” (Bertacchini and Lamieri, 2007, p. 142), transactions become interpersonal. Similar to the market for certifications, “Clientelization represents an actor-level attempt to counteract, and profit from, the system-level deficiencies of the bazaar as a communication network” (Geertz, 1978, p. 30). The presence of this effort rests in turn on a specific function of bargaining: “…the sort of information one needs cannot be acquired by asking a handful of index questions of a large number of people, but only by asking a large number of diagnostic questions of a handful of people” (Geertz, 1978, p. 32). Unfortunately, in the context of ISO certification, this kind of clientelization and bargaining may also question the independence of the auditors and thus the entire system. Boiral (2012) cites one of his informants in a study of ISO certification processes that says the auditors “cannot afford to be extremely strict and cause problems (…) because if an auditor is too strict and causes problems, we can just drop him and look for someone else” (cited in Boiral, 2012, p. 649).

To be able to identify further the pros and cons of the strategy of reaching for certification compared to other types of ES work in the context of the principles of the bazaar economy, this article explores the relations between ES, certification standards, and sport organizations. Similar to conceptual papers in general, the aim is to bridge “existing theories in interesting ways, link work across disciplines, provide multi-level insights, and broaden the scope of our thinking” (Gilson and Goldberg, 2015, p. 128) rather than to present in-depth empirical data or quantitatively measure the effect of various ES initiatives. To flesh out this perspective, the next section introduces a brief review on the growth of international standards and certifications, first in general, then in sports. Then, the case of Formula E's road from start-up in 2014 to ISO20121 certification in 2018 is presented with emphasis of what Bakos (2020,p. 77) call the “pre-institutionalization phase.” Drawing upon this descriptive review, a typology is introduced, which is “a multidimensional view of the target phenomenon by categorizing theoretical features or dimensions as distinct profiles that offer coordinates for empirical research” (Jaakkola, 2020, p. 23). This typology brings pointers to future research that could enrich our understanding of the relation between ES work, certifications, and sport organizations.

## Standards and Certifications: A Brief Overview

To ensure smooth cooperation and competition between countries, sectors, and industries, some form of standardization have always been sought after. According to Brunsson et al. (2012), an organization that has capitalized on this development has been the International Organization for Standardization (ISO). It was founded in 1947 “to promote the development of standardization and related activities in the world with a view to facilitating international exchanges of goods and services, and to developing cooperation in the spheres or intellectual, scientific, technological and economic activity” (Meidinger, 1999, p. 183). Its success is due to four factors. First, the globalization of the economy and transnational supply chains has made it “necessary to foster a certain homogeneity of management systems in order to favor the development of such processes” (Heras-Saizarbitoria and Boiral, 2013, p. 49). Second, this form of auditing reinforces the social legitimacy of organizations through the verification of internal practices “by presumably rigorous, independent and impartial external experts” (Boiral, 2012, p. 634). Third, this homogenization is supposed to ensure the growing diversity of stakeholders by involving third-party bodies in the auditing process (Silva-Castañeda and Trussart, 2016). Fourth, as a result of ever more standards, a possibility to comprise the logic of standards into meta-systems has emerged, which enables organizations to be accountable for their practices beyond technical elements (Uzumeri, 1997).

Despite these benefits, four critiques can be made toward standardization processes. First, the standardization of standardization, so to speak, is hindered by terminological confusion and the lack of a coordinating body. There are technical and non-technical standards, process and outcome standards, and standards inscribed by law and market-driven standards (Brunsson et al., 2012, Nguyen, 2017, Escrig-Olmedo et al., 2019). Second, the codification of the auditing process and techno-scientific values is incommensurable with the social, event-specific, and environmental problems of relevance to the stakeholders (Silva-Castañeda and Trussart, 2016, Naiki and Sakaguchi, 2020). Third, due to the marketization of certifications, Boiral (2012) see this development as an expression of the “degree-purchasing syndrome.” Here, the certification—rather than the aims it is intended to achieve—gets primary attention. In his qualitative study of how certification works, Boiral (2012, p. 644–46) discovered that documents were often “prepared for the impending audit rather than to guide operational activities or meet organizational needs” (p. 644), and actual practices were seldom examined by auditors due to a lack of time. Fourth, the privatization of certification standards open for power games between actors in the field related to the declining capacity and competence among nation-states after the end of the cold war (Heras-Saizarbitoria and Boiral, 2013; Derkx and Glasbergen, 2014; Fransen and Conzelmann, 2014). Timmermans and Epstein (2010, p. 79) therefore underline that:

Depending on the process of standard-setting, standards can imply a lowest common denominator of available options, the power of the strongest party in standardization, a negotiated order among some or all stakeholders, or a confirmation of how things are already done by most parties.

A question that will be explored in next section, therefore, is to what degree do these benefits and downsides of standardization and certification have been addressed in relation to sport?

## Es Work in Sport

Since the early 1990s, sport organizations have paid attention to environmental challenges in a multifarious way. Trendafilova et al. (2018) emphasize three particularly well-researched strands in the literature on ES and sport: sport events, sport facilitates, and the relation between sporting activities and nature. What is noteworthy about these strands, as demonstrated by McCullough and Kellison (2018) as well as by Dingle and Mallen (2020), is that they, for the most part, represent tailormade initiatives to connect global ES issues with local solutions. Less attention has been paid to ES work in relation to international standards or what McCullough et al. (2016) call the third wave of sport environmentalism. Wave one was about developing awareness of environmental issues within their particular context, and wave two came when environmental actions at an organizational level (e.g., team and event) began to merge with league or governing body activities (McCullough et al., 2016, p. 1,053). Wave three concerned strategic planning—often internationally. Typical for this wave is that “certification and process evaluation techniques (…) are implemented to provide stability to strategy and action efforts” (McCullough et al., 2016, p. 1,054). This article argues that the typicalities of the third wave became a norm since the London 2012 Olympics coupled the marketization of standards with corporate partnerships and its own engagement in developing a particular ISO standard.

Unlike early certification adopters like the 2006 Turin Olympics [awarded the ISO 14001, Environmental Management and Audit Scheme (EMAS) certification] (Botta and Comoglio, 2007, Dansero and Mela, 2012), the organizers of the 2012 London Olympics were instrumental in creating the management system that subsequently would become the IOC's preferred certification regime for ES work (Ross and Leopkey, 2017). Way before the 2012 Olympic Games, and well before the bid for the Olympics was placed in 2005, the London Organizing Committee of the Olympic and Paralympic Games (LOCOG) had started to work with ES matters. Fiona Pelham, chair of the team who developed ISO 20121 and who worked closely with LOCOG, explains that:

Existing frameworks at the time were either checklist approaches (limited in their suitability, as it is hard to prescribe generic steps equally applicable across a diverse international event industry) or management systems created for business and focused only on environmental aspects (Pelham, 2011, p. 44).

Therefore, LOCOG and in particular its Head of Sustainability, David Stubbs, collaborated with British Standards (BSI, the UK's National Standards Body, and the first national standards organization in the world) in order to develop a management system designed for events. This standard, which at first became BS8901, brought according to Pelham (2011, p. 45) “attention to the standard and its development process, and supported the work of many event industry individuals passionate for change toward greater sustainability.” A revision in 2009, followed by evidence of use internationally, prompted BSI and the Brazilian National Standards Body (ABNT), to jointly submit a proposal to the International Organization for Standardization (ISO) to transform BS 8901 into a global standard (Walker, 2012, p. 6).

Both BS 8901 and the subsequent standard called ISO20121:2012, Event sustainability management systems—Requirements with guidance for use, take a procedural or process-based management system approach that consists of three phases—planning, implementation, and review—and can be applied by organizers, venues, and suppliers of all types of events (Walker, 2012, p. 7). Basically, it now works like any other ISO standard, which means that the organization following it “must say what it is going to do, how it is going to do it, who is going to do it and by when it is going to get done” (Curcovic and Sroufe, 2011, p. 73). More specifically, the ISO standard includes 29 clauses, which include specific clauses on leadership, supply chain management, event sustainability objectives and sustainable development principles, and statement of purpose and values. As LOCOG was instrumental in operationalizing the forthcoming ISO20121, the result of the Olympics, environmental-wise, confirmed its position in the ISO universe. According to the Commission for a Sustainable London, the Olympic Games watchdog when it comes to ES, “London 2012 has been the most sustainable Games ever” (Commission for a Sustainable London., 2013, p. 2).

Due to this success, the London Olympics' efforts to develop ISO20121 set off an “organizational isomorphism” among other major sporting events (Ross and Leopkey, 2017). The theoretical argument is that organizations in search of legitimacy will at some point begin to become similar due to external demands of what constitutes good governance. In line with how this isomorphism may be coercive, normative, or mimetic (DiMaggio and Powell, 1983, 1991), an example trending toward coercive isomorphism is found in Bakos's (2020) study of ES work at the 2018 Commonwealth Games, where respondents emphasize pressure from LOCOG on other venues to adapt the standard of the 2012 Olympic. On the mimetic side, Rio (2014) which received ISO20121 certification the same year, apparently mirrored the London 2012 Olympics strategy. Besides highlighting the need for “a good management and reporting system to implement effective sustainability measures” (Rio, 2014, p. 38), it says, without further explanation: “The most relevant management system for events is ISO 20121” (Rio, 2014, p. 38). Moreover, the report refers to the London Olympics as the benchmark 16 times, and Rio (2014) sent as part of their pre-Games briefing program 153 observers to experience the Olympic Games to attend 53 official sessions (Rio, 2014, p. 102). Others, as will be demonstrated below with the case of Formula E, are examples of normative isomorphism—finding inspiration from the development of a field-specific norm—but adjusting it to its own capacities (see, e.g., Nichols et al., 2017).

## The Case of Formula E

The Formula E championship has grown considerably in terms of spectatorship, technological development, and commercial interest since its inaugural race in Beijing, China, in 2014. While Formula E can be criticized as it moves around the world to race (responsible for 72% of its carbon footprint^2^ and reinforce the energy-intensive consumption patterns of late-modern society, it has also done more than other sport organizations by actually developing—not only promoting—CO_2_-reducing technology like energy-efficient electric engines and powertrain parts transferable to road cars. For that reason, Krivevski (2018) claims that Formula E has one of the lowest carbon footprints among international sporting events. Even more importantly, the championship has according to Næss and Tjønndal (2021) an approach to “legacy” in ways that mirror LOCOG's David Stubbs' view on what events may bring of ES change: “taken at a large scale, the relatively temporary diversion of resources to deliver the Games can be significantly outweighed by the longterm gains from achieving a sustainable legacy” (Stubbs, 2011, p. 118).

Connected to international accreditations, these ES efforts by Formula E have materialized in two ways. The first came in 2018 when the championship received third-party ISO certification in 2018 and, second, in 2020, when the championship became the first sport with certified net zero carbon footprint from inception. Guided by the recommendations set out by the UN Framework Convention on Climate Change (UNFCCC), Formula E utilized three strategies to achieve this latter award, which is shared with only two other companies in the world: effective measurement of carbon output, prioritizing reducing its footprint and offsetting remaining unavoidable emissions^3^. In other words, it seems to have made it to the top of its aim pyramid in terms of sustainability. What is interesting in the context of this article is that in the *eStory*, Formula E's official “value pamphlet” from 2015 (Wilbaut, 2015), certification is never mentioned—although the focus is on how Formula E as an ecosystem for ES-friendly innovations can improve air quality, provide cleaner mobility solutions (electric ones), and make a positive impact on urban development. In early 2021, with Formula E having raced 50 times in 20 cities and organized six championship seasons across the world, this begs the question of how ISO certification was achieved.

The Formula E's side of the story is told mainly by Senior Sustainability Consultant Julia Pallé. In a master class with Pallé streamed on YouTube in July 2020, facilitated by Factory Berlin TV^4^, she underlined that “don't reinvent the wheel” was a key principle for their environmental work when she joined the company in 2014 from French tire manufacturer Michelin. This meant reading up on the FIA's environmental strategies, scouring UN recommendations as mentioned above, and picking certain known eco-relevant themes that would align with the championship's core issues. Racing in cities, this meant focusing on air quality, energy use, and waste management. To coordinate the efforts to improve on these areas, Pallé says that the next step was to develop a management system to anticipate the expectations in the stakeholder network and find partners that could operationalize tasks. After 1 year of work and racing, Formula E earned some acclaim for its environmental efforts. This opened according to Pallé new doors, such as invitations to join Sport and Sustainability International (which I will return to below), and the green light from Formula E's leaders to expand the ambition from environmentalism to sustainability. This meant including social and economic legacy as well as environmental concern in the equation and strive for what Pallé considered the natural next step: ISO20121 certification, as the aftermath of the London Olympics had proven its relevance to event environmentalism.

At this point, in 2015, the challenge was not only to get ISO certified but sell that specific idea internally. A bottom-up approach was chosen as the ISO certification required all hands on deck and implementation of ES work in all departments. In another interview Pallé, who later became Sustainability Director of Formula E, said that:

The greatest challenge was to create that culture of sustainability within the business. A cultural shift is something really deep and that takes time. You need to be very patient, very stubborn and to truly create synergies. Start building very strong collaborations with your staff and with your exec team. Everyone needs to believe in it. It's not in a couple of days, or even months, that you can create that^5^.

Gradually, Formula E gathered new friends outside motorsports. Pallé mentions in the master class that Formula E was part of the foundation for the UN's Sports for Climate Action framework, such as public endorsements from Greenpeace and invitations to join the French Ministry of Sport's roundtable on sport and sustainability. However, their work was delayed because, in 2015/16, the eco-friendly initiatives were not sufficiently convincing to those who instead criticized the championship's environmental impact from its logistics and intrusion in residential areas (races were held in cities only, on temporary, purpose-built racetracks). Whereas, some events went very well, and major car manufacturers began to enter the championship full time, some situations demonstrate a fallout with the stakeholders and the residents of the city. Most notably, this regards the 2016 season finale in London's Battersea Park. According to the UK branch of SGS (Société Générale de Surveillance), the Swiss-based accreditation company that helped the Rio (2014) Olympics to achieve ISO20121 status and later would do the same with Formula E, the race was:

preceded by a lengthy protest by an action group that claimed motorsport would harm the environment and deprive local people of their tranquility, in spite of the inherently-low emissions and noise levels of the cars participating^6^.

SGS however overlook other reasons to the protests, as the building of race facilities in a historical parkland created restrictions on public use, damaged the greenery, and disturbed local wildlife (including the local zoo, whose animals needed to be moved to make space for construction work) (Smith, 2019, Sturm, 2019). Yet, according to SGS, which describes itself as “the world's leading inspection, verification, testing and certification company” dating back to 1878^7^, this awakening “was pivotal in Formula E's resolution to build on its established environmental management system by seeking official certification of its credentials with the voluntary global standard ISO 20121^8^” Aiming for ISO certification of the management system, by contrast, would in SGS's view “demonstrate that the organization had a complete identification and understanding of its impacts on the environment and local communities^9^” More specifically, SGS sets out to examine the Formula E's “holistic approach to sustainable event management.” This included, SGS writes, the implementation of a waste management system and community engagement projects to ensure social inclusivity.

Pallé, however, underlined in the master class that SGS at first did not realize the full extent of the challenge Formula E was up against with its global traveling and mobile setups. Hence, she says, the two companies collaborated in developing the connection between strategy and operationalization to reach ISO standards. Apart from SWOT analyses and other preparations, Formula E and SGS organized a large stakeholder meeting based on what Pallé names a SMART approach (a concept from management thinking, first coined by Doran, 1981). In this process, local communities, or cities, were, according to Pallé, one of the most important stakeholders to be involved due to urban challenges with air pollution and mobility solutions. Given Formula E's work to address these issues, SGS later published the collaboration as a case study. In the case study report, Pallé says that, apart from the ingenuity required to comply with the ISO management system, there was also a legal issue that would turn out to become far more complex than anybody expected, apparently. As it says in the report:

Not only was there the documentation preparation and review and proof gathering, but the necessity for development of a Legal Register, which records all legislation directly or indirectly applicable to operations and activities, in every location an event is held. This is made more complex as, in many cases, events held from year to year may be in different locations within the same country, each with its various requirements and characteristics (SGS, 2018, p. 3).

According to Pallé, reaching for ISO certification meant altering the coordination of the FE business: “We were successful because we gathered all of the departments of the business around the table—communications, event managers, procurement—and identified key issues and carried out contextual analyses of those issues” (cited from Campelli, 2018). Furthermore, a specific ISO 20121 Working Group was, according to Campelli (2018), set up “with a dedicated representative from each department, with additional working groups built around topics like community engagement, waste management and supply chain.” The final push for ISO certification came in 2017 and 2018, going for third-party certification (the most highly rated certification) where onsite auditors according to Pallé at the master class examine whether you can prove, document, and demonstrate that you do everything that is stated by the management system for ES work. Apparently, it worked, as Formula E received the ISO certificate in August 2018, 1 month after the New York races.

In early 2021, Pallé leads the central contact team, which has a representative in each racing team to ensure coherence in the sustainability policy. These moreover work together with the rest of the team as well as the rest of Formula E to develop the sustainability practices^10^. In achieving the net zero carbon footprint achievement, for example, Formula E worked with Quantis, a life cycle assessment consultancy that also aided the 2016 Rio Olympics in creating an environmental calculator^11^, to calculate the overall footprint of the championship. To offset this Formula E's emissions from the past six seasons, the championship has, alongside “traditional” projects like optimizing transport and logistics, invested in Gold Standard (GS, a non-profit organization established by the World Wildlife Fund, SouthSouthNorth and Helio International in 2003 and HQ'ed in Switzerland) and Verified Carbon Standard UN projects in line with the UNFCCC's Clean Development Mechanism. Among the projects, Formula E lists the building of 15,555 methane digesters on the Chinese island of Hainan in which organic matter, including manure and waste, is anaerobically degraded into methane gas through microbial action—saving 53,000 tons of CO_2_ per year^12^ Again, the market rules: According to Gold Standard's own help desk, it sells Verified Emission Reductions (carbon credits) on behalf of the project developers running these projects. Project developers can then sell their credits at any price above the minimum price based on the Fairtrade carbon credit pricing model^13^.

**Figure 1:**
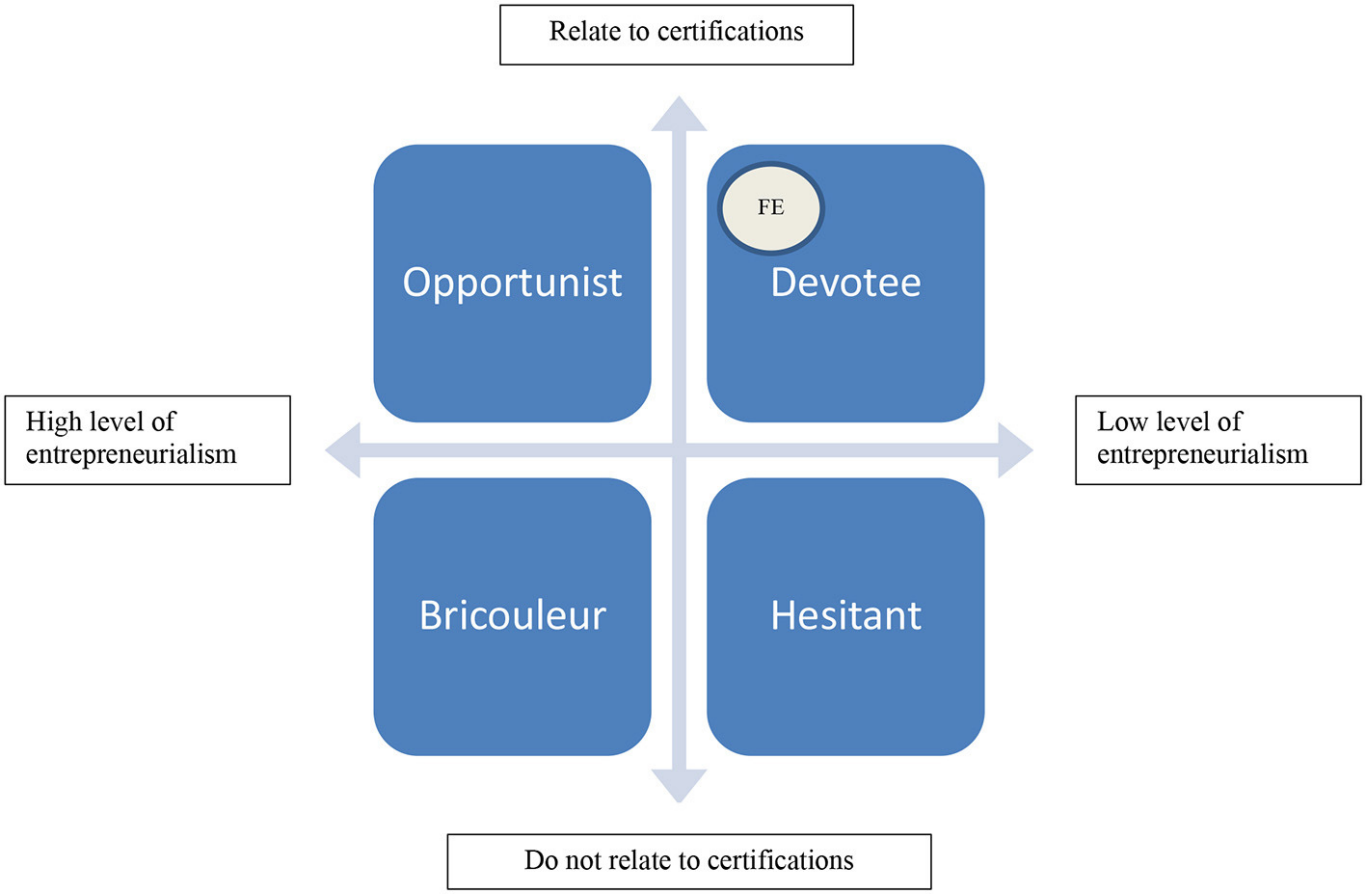
A conceptual typology of ES work related to certification.

This carbon market in which both ISO-certified companies and carbon credit brokers are placed is in other words filled with actors collaborating through what Pratash and Potoski (2006) name “green clubs.” According to Foubert's study of GS diffusion (2010, p. 22), such clubs in particular provides “its members with several advantages, such as the possibility to build organizational reputation thanks to the established reputation of the club and its sponsors.” Affiliation with these green clubs moreover enable organizations to use the certification as a branding mechanism and access a network of what Foubert (2010, p. 22) call “environmentally pro-active organizations, which has more visibility than an organization left alone.” In this way, Formula E-SGS relationship and its ES strategy is an example of the argument that Brunsson et al. (2012) make from the impact of standardization on organizational structures and administrative procedures. Interestingly, the case example of Formula E as “devotee” (see Figure 1) shows that this devotion or desire to be a member of the club does not have to be there from the beginning. It may equally well emerge when the organization is scaling up or increasing its complexity to further its mission, thus *investing* in certification as a business strategy. Supporting this view is the publication on Formula E's own website—along the news of having maintained its ISO20121 certification for 2020—of a quote from Ana Inacio, SGS Auditor: “Formula E has been embedding sustainability season-on-season—exploring new initiatives, engaging with local communities and suppliers, and applying robust impact assessments. It is taking the lead in sustainable motorsports and setting a new benchmark for the industry^14^.”

## A Conceptual Typology

Earlier in this article, we discussed how the London 2012 Olympics was the starting shot for sporting organizations in acquiring international certifications as evidence that their environmental policies are credible. Our subsequent exploring of Formula E as one of the key proponents for continuing this approach, generated an impression of it as a *devotee* to the ISO solution for demonstrating ES credibility (see the round spot in Figure 1 for a more precise location). Put short: These organizations, as exemplified not only by Formula E but also by the Rio Olympics and Manchester United FC's multiple ISO certifications in 2012 where SGS again was involved^15^, go all-in to make their investment worthwhile in a certification market that show signs of becoming what Geertz (1978) and others have named a “bazaar economy.” However, as addressed by Pallé, Formula E also had to engineer solutions on how to apply certification criteria to Formula E due to the lack of knowledge at SGS. Meanwhile, many sport organizations, especially smaller ones, have chosen to tackle ES challenges in other ways, and by other means. To explain the use of this term, I will use the story of Formula E above as a signpost for a comparative typology whose aim is to enable broader examinations of the relationship between ES issues, certification standards, and sport organizations. The typology thus represents possible approaches to certification in a bazaar economy. The following examples are consequently not an exhaustive list of cases fleshing out each approach, but only serves as indications of ways to approach the topic of ES work and certifications in sport.

Down to the bottom left, we find *bricoleurs*. A key issue for these organizations is to combine entrepreneurship, which in ES work has long been encouraged (Ratten, 2019), with acknowledged practices. The question is whether universal standards help or hinder them in this work. In the UK, empirical research demonstrates that football clubs like Manchester City FC and events like FA Cup Final have actively searched to reduce their “ecological footprint” since years back (Collins and Flynn, 2008, Collins and Cooper, 2017). A related approach is offered by Kellison and Hong's (2015) study of building of eco-friendly sport facilities. In their sample of 25 pro-environmental sport facilities spanning four continents, all sharing the same goal, the researchers found considerable diversity and innovativeness in the interaction between designers, owners, stakeholders, and the general public. A third example is that the New York Yankees has hired sport's first environmental science advisor in the shape of Dr. Allen Hershkowitz in 2019 (Campelli, 2019). Although being lauded for being instrumental in the Yankees' carbon offsetting projects like delivering cookstoves in economically deprived regions in Africa, thoughts on ISO certification are nowhere to be found in Hershkowitz' work. This despite the fact that Hershkowitz co-founded and now chairs the sustainability network Sport and Sustainability International (SandSI), established in 2016, where Formula E's sustainability boss Julia Pallé in 2018 was elected president.

For the *hesitant*, the worry is lack of transparency, efficiency, and cost issues related to certification processes (see, e.g., Andersson, 2016). For example, a study of ISO14001, a standard designed to aid companies toward managing environmental issues and awarded to the 2006 Turin Olympics, showed that despite vast implementation in the US and the UK, “it is not connected directly enough to environmental performance” (Curcovic and Sroufe, 2011, p. 73). Moreover, the mere cost can be high or low, depending on the characteristics of the organization. According to Schuurman (1997), whose study was based on a sample of 1880 companies in the USA and Canada, the average total implementation cost for ISO14001 certification depended on turnover, number of employees, and classification. For a company with 11–25 million USD in turnover and 150–500 employees, this meant a cost of USD 121,000 (Schuurman, 1997, p. 25). More recently, NQA, a global certification body based in the UK, informed in 2017 that “The exact quote given [for ISO certification] will vary based on the certification body's rates, your organization and the standard you're seeking certification for^16^” A similar stance was taken in July 2020 by ISO Update, an information site on ISO-related issues. Although not willing to estimate the total cost for certification, it still says that “You can expect an average ISO Certification to cost around $3,000–$5,000 annually^17^” Other suppliers of certifications, like ISO Accelerator in the UK, offer ISO14001 Environmental Management System certification “from just £695” and in addition deliver “fast-track certification,” which “typically takes just 2 working days^18^”This is possible, ISO Accelerator claims, due to a different business operation than other certification bodies^19^.

For organizations taking an *opportunist* approach, the assessment of the value of choosing one standard over the other, or combining certifications, is key to their decision. These combinations, moreover, may be motivated by economic or environmental considerations. ISO Update, for instance, claims that “While certification may seem expensive, the opportunity cost of no certificate is much greater. Consider the number of deals you've lost without certification, the level of inefficiencies you run from wasteful practices and processes, and the associated long-term costs^20^” This development runs the risk of turning into a self-fulfilling prophecy, as companies with multiple certifications, according to Wiengarten et al. (2017, p. 131), are “significantly better performers with regard to environmental and occupational health and safety compared to companies without multiple certifications.” This “the more the merrier” kind of thinking may create a shopping spree of various certifications as the diversity of the certification market enables event organizations “to choose based on affordability and the potential to improve their image” (Andersson, 2016, p. 25). For example, in their study of pro-environment sport facilities, Kellison and Hong (2015, p. 254) refer to Leadership in Energy and Environmental Design (LEED) certification as desirable, as it was “one of the earliest and most prominent of these certification systems.” As a final example substantiating this category, Naiki and Sakaguchi's (2020) uses the struggle behind the delivering of sustainable agricultural and fishery products to what should have been the Tokyo 2020 Olympics to discuss whether local certification programs soon may outperform global ones.

## Discussion, Conclusions, and Implications

By using Formula E's road to ISO certification and net zero carbon footprint as case example, this article has discussed the relation between ES work in sport organizations and certification standards. Is striving for certification a detour or a gamechanger for eco-striving sport organizations that seek to reduce “organizational hypocrisy”? The answer to that depends on what sport organizations are ready to invest in a certification, to what degree the organization is equipped, either organizationally or by disposition, to address conflicting logics, and whether the organization is ready to operate the market in order to maintain the certification. In particular, it depends on whether the organization is capable of handling the logic in a system where, similar to the bazaar, “little is packaged or regulated, and everything is approximative,” making the possibilities for bargaining along non-monetary dimensions enormous (Geertz, 1978, p. 31).

Thus, one aspect is necessary to address: Whatever category a sporting organization may belong to in the typology developed above—the opportunist, the devotee, the hesitant or the bricoleur—they are all part of an international development where the certification of ES standards is embedded in what appears to be a bazaar economy. In lieu of state coordination, the changing role of civil society, and the proliferation of private actors in global governance (Glasbergen and Schouten, 2015, p. 86), working the bazaar economy of certifications rely—like in Geertz' original study (1978)—on making decisions on the basis of distorted knowledge and information asymmetry. A final example: For a sporting company desiring to become certified net zero carbon footprint since inception, for example, numerous agencies are ready to offer their certifications like vendors in a bazaar. Whereas Formula E worked with Quantis, as mentioned above, the Formula E Mahindra Racing Team chose the ALLCOT Group to make it the first FIA World Championship entrant to be certified net zero carbon footprint^21^. This lack of coordination combined with sub-markets, along with the devastating effects from the Rio Olympics on the environment despite its ISO certification (Malhado and Araujo, 2017) and high-profile marketing from Formula E, surely evoke suspicions of “greenwashing.” According to the strongest critics, Formula E is nothing but hypocritical in its sustainability vision (Ariès, 2018), and Helmut Marko, a senior consultant to the Red Bull Formula 1 teams, said in 2020, “Formula E is for us only a marketing excuse from the automotive industry^22^.”

Nevertheless, whether ISO certification is a detour or a gamechanger—or perhaps even a greenwashing exercise—also depends on what the ES aims for the organization are. In the case of Formula E, the business impact of the certificate was an obvious pull factor^23^ However, it is not certain that all organizations can defend this kind of investment toward their stakeholders. Selecting an alternative to certification may conversely generate an impression that the organization does not take ES seriously, as legitimacy comes from adherence to the system and approval from its auditors. At the same time, in several cases, the freedom to create innovative solutions outside certification regimes seems far greater than doing what is necessary to become a member of the “green club” and turning ES work into a product whose cost–benefit ratio is determined by market forces and private initiatives. In fact, going for certification may strengthen the impression of organizational hypocrisy by making ES promises that are hard to keep and being accused of “buying” green credentials. Rather than being an example of conventional “decoupling”, which happens when “organizations abide only superficially by institutional pressure and adopt new structures without necessarily implementing the related practices” (Boxenbaum and Arora-Jonsson, 2008, p. 81), it might signal “organized hypocrisy.” Instead of implying that talk, decisions, and actions are decoupled, they are “coupled in a way other than usually assumed” (Brunsson, 2006, p. 115–116).

The implications for researchers are thus that to address the pros and cons of seeking ISO certification compared with other ES initiatives, they should go deeper into what appears to be a bazaar economy of ISO certifications and examine empirically and comparatively the road from idea to certification and finally its effects. For instance, what if clientalization becomes a threat to the system because it “partitions the bazaar by creating relationships between buyers and sellers based on ‘those in the know,' meaning that new arrivals to the bazaar may be far more exposed than regular participants to the vagaries of information flows” (Depledge and Dodds, 2017, p. 150)? To provide a starting point for exploring this diversity, this article offers a conceptual typology that, rather than being an exhaustive explanation of how sport organizations relate to the ISO universe, is a map of possible positions that can be used to compare practices.

## Data Availability Statement

The original contributions generated for the study are included in the article/supplementary material, further inquiries can be directed to the corresponding author/s.

## Ethics Statement

Ethical review and approval was not required for the study on human participants in accordance with the local legislation and institutional requirements. Written informed consent for participation was not required for this study in accordance with the national legislation and the institutional requirements. Written informed consent was not obtained from the individual(s) for the publication of any potentially identifiable images or data included in this article.

## Author Contributions

The author confirms being the sole contributor of this work and has approved it for publication.

## Conflict of Interest

The author declares that the research was conducted in the absence of any commercial or financial relationships that could be construed as a potential conflict of interest.

## Footnotes

^1^Cited from “Event sustainability management - ISO 20121 passes 2012 Olympic Games test”, iso.org, January 9, 2013. Retrieved from: https://www.iso.org/news/2013/01/Ref1690.html.

^2^“Formula E: achieving net zero carbon footprint”, formulae.com, September 21, 2020. Retrieved from: https://www.fiaformulae.com/en/news/2020/september/three-steps-to-net-zero).

^3^“Formula E claims first sport to have net zero carbon footprint”, September 21, 2020. Retrieved from: https://www.racetechmag.com/2020/09/formula-e-claims-first-sport-to-have-net-zero-carbon-footprint/.

^4^“Driving the Future: Masterclass with Julia Pallé (Formula E),” Factory Berlin TV, July 22, 2020. Playtime 58:15 minutes. Retrieved from: https://www.youtube.com/watch?v=D-upisARrfkandfeature=youtu.be.

^5^“Julia Pallé: Driving Formula E's Sustainability Strategy”, Medium.com, November 8, 2020. Retrieved from: https://charlotte-hook.medium.com/julia-pall%C3%A9-driving-formula-es-sustainability-strategy-a97f8e546097.

^6^“Formula E achieves demanding event sustainability certification”, SGS.com, September 2, 2018. Retrieved from: https://www.sgs.co.uk/en-gb/news/2018/09/formula-e-achieves-demanding-event-sustainability-certification.

^7^“ABB FIA Formula E championship receives ISO20121 certification from SGS”, SGS.com, August 20, 2018. Retrieved from: https://www.sgs.co.uk/en-gb/news/2018/08/abb-fia-formula-e-championship-receives-iso-20121-certification.

^8^“Formula E achieves demanding event sustainability certification”, SGS.com, September 25, 2018. Retrieved from: https://www.sgs.co.uk/en-gb/news/2018/09/formula-e-achieves-demanding-event-sustainability-certification.

^9^“Formula E achieves demanding event sustainability certification”, SGS.com, September 25, 2018. Retrieved from: https://www.sgs.co.uk/en-gb/news/2018/09/formula-e-achieves-demanding-event-sustainability-certification.

^10^This was explained by Palle in conversation with Professor Simon Chadwick during the ‘Webinar: Sport and sustainability - lessons from Formula E', hosted by Emlyon Business School on November 18, 2020.

^11^The entire case study of the 2016 Rio Olympics was retrieved March 1, 2021, from: https://quantis-intl.com/about/our-work/case-studies/#modal-448.

^12^“Formula E: achieving net zero carbon footprint”, formulae.com, September 21, 2020. Retrieved from: https://www.fiaformulae.com/en/news/2020/september/three-steps-to-net-zero.

^13^“How are carbon credits on the marketplace priced?” Gold Standards Help Desk, November 30, 2020. Retrieved from: https://goldstandardhelp.freshdesk.com/support/solutions/articles/44001966875-how-are-carbon-credits-on-the-marketplace-priced.

^14^“Formula E maintains ISO 20121 certification for sustainable events,”, fiaformulae.com, January 14, 2020. Retrieved from: https://www.fiaformulae.com/NEWS/2020/January/formula-e-maintains-iso20121.

^15^“14001 first for Manchester United”, Transform.iema.net, February 23, 2012. Retrieved from: https://transform.iema.net/article/14001-first-manchester-united; see also “Event sustainability management - ISO 20121 passes 2012 Olympic Games test”, iso.org, January 9, 2013. Retrieved from: https://www.iso.org/news/2013/01/Ref1690.html.

^16^“What are the Costs of Third Party Certification?” Nqa.com, July 20, 2017. Retrieved from: https://www.nqa.com/en-gb/resources/blog/july-2017/costs-of-third-party-certification.

^17^“The Cost of ISO Certification”, ISOupdate.com, July 20, 2020. Retrieved from: https://isoupdate.com/general/the-cost-of-iso-certification/.

^18^Read more about pricing and the like at ISO Accelerator at: https://www.iso-accelerator.co.uk/learn-more/how-much-does-it-cost.

^19^Read more about ISO Accelerator at: https://www.iso-accelerator.co.uk/about/faqs.

^20^“The Cost of ISO Certification”, ISOupdate.com, July 20, 2020. Retrieved from: https://isoupdate.com/general/the-cost-of-iso-certification/.

^21^“Mahindra Racing First Formula E team and FIA World Championship Entrant to be certified net zero carbon footprint since inception”, mahindraracing.com, February 4, 2021. Retrieved March 10, 2021, from: https://www.mahindraracing.com/team-certified-net-zero-carbon-footprint/.

^22^“Formula E boss: Electric series ‘flattered' by criticism from F1”. *Autosport*, July 21, 2020. Retrieved from https://www.autosport.com/fe/news/150747/formula-e-boss-flattered-by-f1-criticism.

^23^“ABB FIA Formula E championship receives ISO20121 certification from SGS”, SGS.com, August 20, 2018. Retrieved from: https://www.sgs.co.uk/en-gb/news/2018/08/abb-fia-formula-e-championship-receives-iso-20121-certification.
